# Patients help other patients: Qualitative study on a longstanding community cooperative to tackle leprosy in India

**DOI:** 10.1371/journal.pntd.0008016

**Published:** 2020-01-13

**Authors:** Seong Hye Jung, Hee Won Han, Hyeonseok Koh, Soo-Young Yu, Nobutoshi Nawa, Ayako Morita, Ken Ing Cherng Ong, Masamine Jimba, Juhwan Oh

**Affiliations:** 1 Department of Medicine, Seoul National University College of Medicine, Seoul, South Korea; 2 Department of Nursing, Seoul National University College of Nursing, Seoul, South Korea; 3 JW LEE Center for Global Medicine, Seoul National University College of Medicine, Seoul, South Korea; 4 Institute of Education, Tokyo Medical and Dental University, Tokyo, Japan; 5 Department of Global Health Promotion, Tokyo Medical and Dental University, Tokyo, Japan; 6 Department of Community and Global Health, Graduate School of Medicine, The University of Tokyo, Tokyo, Japan; Hospital Infantil de Mexico Federico Gomez, UNITED STATES

## Abstract

**Background:**

Although leprosy is portrayed as a disappearing disease, leprosy affected persons in India are still suffering massively. Even further, nearly 60% of the world’s newly detected cases are appearing from India alone. The problem has exacerbated due to the drastic decrease of global funding after India’s official declaration of ‘elimination’, which did not foster the actual pain of patients beyond prevalence. Leprosy patients have hardships in their lives due to disabilities, stigma and poverty; thus, they require sustained, continuous care even after release from treatment. Yet, current interventions mostly have a vertical, short-term approach, not showing much progress in lightening the burden of leprosy. In contrast, Little Flower Hospital Community (LFHC) in India has been remarkably providing holistic care for thousands of leprosy patients for 35 years. However, there has not been any research conducted to uncover the underlying factors of this longstanding leprosy control model. Therefore, this research explores the in-depth contextual attributes of this hospital community that has been able to successfully provide sustainable care for a long time even without excessive external funds.

**Methods and findings:**

This qualitative research used a grounded theory approach, involving 28 in-depth interviews of 11 patients, 13 workers, and 4 board members from the hospital. The interview data were inductively analyzed to examine the contextual factors of the hospital’s sustainability. Open coding, axial coding and selective coding were conducted, and Glaser’s Six C’s model was used to create a theoretical model of the sustainability of LFHC. The fundamental cause of the sustainability was the leprosy patients’ strong craving for life with dignity, despite the isolation from the society. The desire resulted in a bottom-up formation of a ‘consumer-provider cooperative’, where patients mutually support each other with basic treatment learned from experience. The profits earned from the patients’ occupational efforts such as dairy farming, cover the costs needed to manage the hospital community, which contributes to economical sustainability. Social sustainability was established through the holistic care including psychosocial, educational, medical, and residential support. The wholesome care socially rehabilitated the patients to be included in the society with satisfaction, social justice and social cohesion. The main limitation of this study is that this study cannot be generalized due to the nature of Grounded Theory based study.

**Conclusions:**

This study investigated the determinants that made LFHC sustainable, and the findings suggested the importance of forming a cooperative community and implementing social rehabilitation for sustainable leprosy control. More exploration on transferring this model to other leprosy colonies will have great impact in maintaining sustainable care for leprosy patients. Furthermore, this research may highlight the importance of sustainable development in policies targeting neglected tropical diseases beyond leprosy as well.

## Author summary

A typical day for a leprosy patient starts like this. One wakes up and discovers that the ulcer on his left foot has worsened over a few days. Strong odor coming from the wound is disturbing and skin continues to deteriorate but he can’t visit the clinic because it is too far from home and also because he does not have enough money. He leaves the house to beg for money on the streets. As usual, people try to avoid being close to him and stares at him with fear. A typical day for a leprosy patient from Little Flower Hospital Community (LFHC) goes like this. One wakes up from the housing provided by LFHC and discovers that the ulcer on her left foot has worsened over a few days. She visits the hospital nearby and gets her wound checked up by the nurse. She leaves the hospital to work at the dairy farm, which is a job provided by the LFHC. The money that she earns is used to support her and the hospital both. As usual, people in the community greet her with a smile. LFHC created a vast difference in the life of the patient, meeting one’s holistic needs. This research explores how LFHC was able to provide appropriate care for leprosy patients and sustain itself for 35 years. The possibilities of implementing the sustainable model of LFHC to marginalized people in the rest of the world are noteworthy.

## Introduction

Since WHO had launched a goal to eliminate leprosy as a public health problem by the year of 2000, prevalence of leprosy declined substantially to achieve the number of 1 case or fewer per 10,000 population at the global level [1, 2]. The goal was also achieved in India, declaring the elimination of leprosy with a national prevalence rate (PR) of 0.96 per 10,000 population on December 31^st^, 2005 [3, 4]. This official elimination declaration was an important milestone for leprosy control; however, contradictory situation is proceeding towards unfavorable circumstances for India. The declaration of elimination induced a massive decrease in global funding and reallocation of resources for leprosy programs in India [5]. Due to limited resources, leprosy specialized services in India had to be integrated with the general healthcare system, which diverted attention away from leprosy [4]. Thereafter, it has been difficult for the government to sustain leprosy control activities. India still accounts for nearly 60% of all the world’s incidence in leprosy and it seems that numbers are not going to decline anytime soon due to reduced government support [5].

One of the barriers to early diagnosis and effective control of leprosy is stigmatization and discrimination against leprosy affected persons (LAPs). Leprosy has been portrayed as a form of divine punishment in many cultures and religions [6]. Moreover, leprosy progress towards severe physical sequelae such as blindness, numbness and amputation of limbs [7]. Because of stigma as well as disabilities, leprosy patients require continuous aftercare and an inclusive society to live in [7]. The LAPs, which include patients who have leprosy, who have achieved the complete cure of leprosy and the family of leprosy patients, also need constant support so that relapse or new contamination can be prevented. WHO has acknowledged the importance of sustainable care in leprosy and presented the need of integrated strategies, stronger surveillance and social inclusion of leprosy patients in the ‘Global Leprosy Strategy 2016–2020’[8]. Several other researches emphasize sustainability as well, proposing the challenge of long-term leprosy control for the future [4, 7, 9].

Although a myriad of studies stresses the significance of sustainable support for the patients’ well-being regarding the etiology of leprosy, most of the interventions for leprosy care are based on a vertical, short-term approach. Since the recommendation of multidrug therapy (MDT) including rifampicin, dapsone and clofazimine for every patient was introduced, almost all of the strategies for leprosy targeted on the provision of MDT, focusing merely on the clinical aspect of the disease [10]. There was a widespread optimism for the elimination goal ever since the indicator of ‘the prevalence of patients registered for treatment’ has started to decrease substantially after the application of MDT. However, this indicator has not been embracing the status of the discharged people still left with disabilities and deformities [11]. There needs to be a new focus on the actual suffering of leprosy patients, including restrictions that they face due to disabilities after release from treatment. To ensure them a better and healthier life, it is important to provide a social environment that can supply continuous care, rather than just medicine.

Little Flower Leprosy Hospital (LFH), which is located in Bihar, India, has been serving thousands of LAPs for 35 years, including over a decade after the large funding cuts with the declaration of leprosy elimination in India. Founded in 1981 by a Christian priest, the goal of LFH was to keep leprosy patients from being left behind in society. The founder hoped leprosy patients would be able to make a living on their own with dignity. It has been providing the appropriate care needed for patients, the patients’ families, and the past patients who are cured but still isolated from the society due to stigma and deformities. There has already been many researches recognizing the stigma of leprosy, highlighting the need of reducing stigma through counseling or contact interventions [12, 13]. Beyond tackling the problem of stigma, LFH is distinct in that it is ‘a favorable community to live in’, where LAPs can live a daily descent life by overcoming their disabilities with their own power. However, there has not been any research conducted to reveal the essences of success for the sustainable community-based rehabilitation that aims at improving well-being and quality of life in the LAPs. Therefore, this study aims to explore the reason why this hospital has been able to sustain itself until now, through exploring the innermost feelings of leprosy patients toward the hospital, and to establish a sustainable development model that can provide long-term care to change the lives of leprosy patients.

## Materials & Methods

### Study design

An inductive approach of Grounded Theory using semi-structured interviews was undertaken to explore the experiences and thoughts of patients, workers, and board members of LFH. Grounded Theory, a method of qualitative research developed by Glaser and Strauss, is known to be suitable for studying social interactions and behavior, especially in areas of research that have not yet been deeply explored [14]. Therefore, the method of Grounded Theory is used in this study to explore the reason why this hospital has been able to sustain itself until now.

### Study sites

The studies were conducted in Raxaul, Bihar, which is a district located in India near the border of Nepal. India is still one of the 22 ‘global priority countries’ which accounts for 95% of the global burden of leprosy [5, 15] and Bihar is one of the poorest districts in India, where one fifth of the whole leprosy patients of India have been forming an endemic colony [16]. Thus, this area is characterized by leprosy patients begging for a living, and by communities that lack basic services such as education, medical treatment and sanitation.

LFH, playing a big role in caring for these patients in Bihar, was the participating study site of this research. Currently, the hospital is surrounded by Little Flower Hospital Community (LFHC) inhabited by more than 1,000 people. There are about 2,000 more LAPs from other leprosy colonies who came to the hospital as well. Collectively, LFHC has been able to support more than 75,000 people for more than 35 years.

### Study participants

The study population was composed of both genders, ages ranging from early twenties to late seventies. A varying relevance to the LFHC was included, ranging from patients living in the colony for 35 years to patients that have only been admitted for 1 week. The inclusion criterion was to have an experience with the LFHC, and willingness to participate in the study. Participation for the interviews were voluntary, without any financial obligations or rewards. The interviewer met with two of the board members, who were interviewed during the study, to explain the purpose of this study and gained consent from the hospital 6 months before interviews took place.

Study participants were purposefully recruited on the basis of their experience with the hospital, and were divided into three groups: 1) patients group (11 participants, labeled as P1 ~ P11), 2) workers group (13 participants, including those who can also be current patients, former patients or family members of patients, labeled as D1~D3 for dairy farm workers, K1~K4 for scarf making workers, W1~W3 for wound patch producers, N1~N3 for nurses and wound dressers), and 3) board members group (4 participants, labeled as BM1~BM4). The characteristics of the participant interviewees are described in Table 1.

**Table 1:** Socio-demographic characteristics of participants (n = 28).

Characteristics	Number of interviewees	Characteristics	Number of interviewees
**Sex**		**Relation with leprosy**	
Female	8	Current or past patient	15
Male	20	Family of leprosy patients	7
		No relation with leprosy	6
**Age**		**Occupation**	
20s	3	Working in LFHC	21
30s	4	Working outside LFHC	3
40s	6	No occupation	4
50s	7		
60s	7		
70s	1		
**Number of children**		**Resident**	
No children	6	Born and living in LFHC	4
1–3 children	15	Living in LFHC	13
4–6 children	7	Living outside LFHC	11

### Ethical approval

This study was approved by the Seoul National University Hospital Institutional Review Boards (IRB). All study participants were informed about the purpose and objectives of the study and signed a voluntary consent form before participating in the interview. Compensations for the interviews were not provided. All the transcripts and records of the interviews were kept confidential and was only accessed to the three researchers.

### Data collection

Semi-structured individual interviews were conducted face to face for an average of 40minutes by the three researchers between July 21^st^ and July 29^th^, 2018. To follow the inductive approach of Grounded Theory, interviews were conducted with flexibility, based on the broad pre-made interview protocol [14]. The patients were asked questions related to their experience of being admitted at the hospital, and their satisfaction with the services and physical surroundings. The workers were asked about their motivation for starting to work at LFHC, and their change in life before and after working. This study intended to pursue deep understanding of this community through in-depth interviews of the board members, and thus requested to describe the specific service provision methods and the hidden factors regarding the hospital’s sustainability and success.

The interviews were conducted using the local language Bihari, and words were translated to the interviewers by the interpreter, who is a physiotherapist with vast knowledge and experience regarding leprosy, speaking both Bihari and English. The physiotherapist did not have a direct working relationship with the hospital but had visited the hospital several times before while working for an NGO. Confirming questions were continuously asked to the interpreter to reduce misinterpretation of the meanings of specific quotes. Interviews took place at comfortable places for the interviewees; at the hospital wards, workplaces or patients’ houses, which are all places that patients are very much familiar with. The research team concluded the interviews when no new themes emerged, which indicated saturation of the context. All the interviews were recorded with an audio recorder with permission, then transcribed and translated into English by the research team accompanied by the interpreter after each session.

### Data analysis

**Fig 1:**
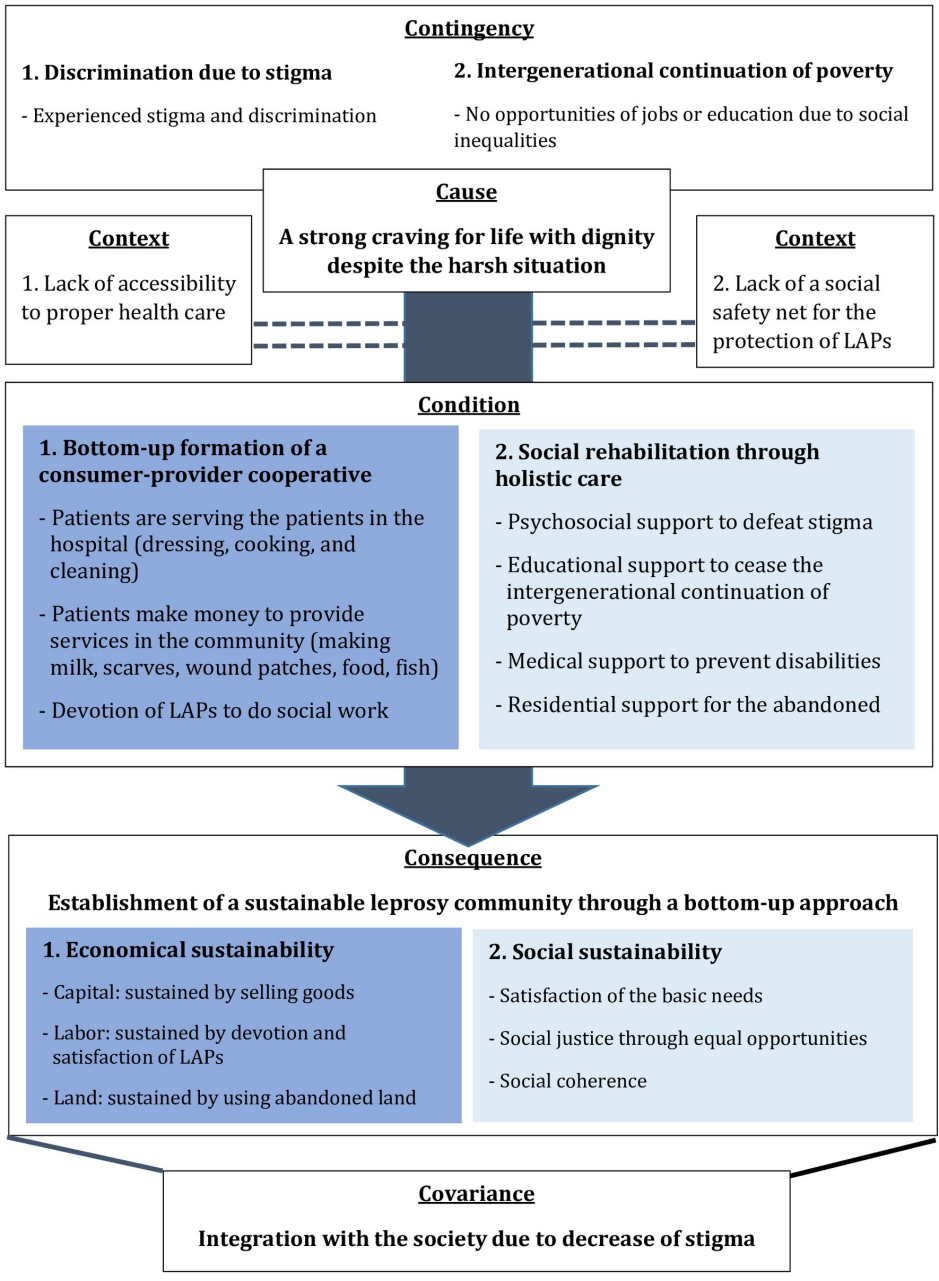
The theory of how Little Flower Hospital Community is sustainable using Glaser’s Six C’s model (Cause, contingency, context, condition, consequence, covariance). Fig 1 explains the factors contributed to the formation of LFHC. Cause, condition, consequence shows sequential process in which LFHC went through in order to reach its current condition. Contingency shows the background factors and Context elaborates the social environment factors which influenced this process.

Data was analyzed based on Grounded Theory, using an inductive approach to develop concepts and construct categories [14]. Analysis began with multiple thorough reviews of all the transcripts containing verbatim. Line-by-line analysis was conducted to generate ‘codes’ [17]. The coding process involved identification of the significant sentences and discovering the key concepts from the data [18]. The emerging codes were constantly compared to previous codes and thus produced a higher level of abstraction called ‘concepts’, further leading to ‘categories’ [14]. Then, selective coding was conducted to integrate and refine categories to make a theory [19]. For the final results, we categorized the themes and created a theory of the sustainability of LFHC by using Glaser’s Six C’s model [17, 20]. This model of theoretical coding consists of *causes*, *contingencies*, *context*, *condition*, *consequences* and *covariance*. *Cause* is the fundamental reason that induces the *consequence*. *Contingency* is defined as the state that must be present to provoke the *cause*. *Context* is a term for the specific circumstances related to the *condition*. *Condition* is the component that must exist for the *cause* to result as the *consequence*. *Consequence* is the result that occurs due to the presence of all the aforementioned four factors. *Covariance* is a term for the phenomena that appears with the consequences [20]. Each of the components are explained in *Fig 1*, with corresponding subsection numbers. The following result section contains quotations that particularly highlights the important concepts derived from interviews conducted.

## Results

### Cause

The participants showed enthusiasm and liveliness while talking about their lives with jobs and education. LAPs wanted to escape the unequal society through a chance of earning money to support their own family. They were willing to have the opportunity to develop their potential and abilities without being limited even before trying.

*What are the leprosy people demanding*? *They are demanding good life*. *They were supposed to live a good life*, *but it was deprived*, *taken away by the society*. *… The meaning of ‘good life’ is not a rich life*. *They just want a good life*, *where their children can get education*, *they have a capacity to buy good food*, *wear simple dresses*, *and have a little savings so that they don’t have to get hardship if something happens to them*. *They want to be secured*. . . . [BM1, female]

### Contingency

Leprosy patients and even the children and family of the patients were born to live in a society against them, and thus they were deprived of the opportunity for livelihood. Such a situation made them even more desperate for life with dignity.

#### Discrimination due to stigma

After being diagnosed with leprosy, patients were abandoned by their families, without a place to go. The society did not allow patients to live in the same village, use the same utensils, or be inside the same shop. When the patient’s families tried to offer support, the society isolated the whole family.

*When I went to the tea shop to drink tea*, *the owner told me to leave immediately*. *He yelled*, *“Take the tea and go out*!*”*. *He said that other customers will see my disability and not come back to the shop*. *Also*, *shoe technicians in the market didn’t want to help me*. *Sometimes when my footwear gets old and needs repair*, *I wanted to fix it but the shoe technicians in the roadsides wouldn’t want to touch it*. [P3, male, leprosy patient with body disfigurement]

*One time*, *people from the village organized a plan to give me poison and kill me*. *It was due to stigma*. *If I live in the village*, *people thought that the disease might spread*. *And also*, *due to social stigma*, *all my family members couldn’t get married*. *Just because of my disease*. [P9, male, leprosy patient with amputation]

*The first time when I was diagnosed with leprosy*, *I couldn’t say a word because of shock*. *I felt that I should jump in front of a running train and die*. *I also thought of jumping into the pond and killing myself*. *I hated myself*. [P10, male, leprosy patient with body disfigurement]

#### Intergenerational continuation of poverty

The physical symptoms make it difficult for LAPs to work and the visibility of the disability forbids them from being hired. LAPs are not able to develop skills for jobs because they are not provided with any education opportunities. The lack of economic power induces an intergenerational continuation of poverty, depriving the opportunity for the children of patients to leave the cycle of poverty as well. Generations of leprosy patients resorted to begging for survival just because they or their family member had leprosy.

*Previously*, *when I was living in another colony*, *I had a lot of unhappiness*. *I had to go begging*. *Our family did not have a good house or electricity*, *or even good water to drink*. *My mother and father would go begging*, *so I also had to go to another village for begging*. [W3, female, family of leprosy patients]

*A lot of people with leprosy beg in the streets for a living*. *In the cities*, *you can see patients begging for their livelihoods and nobody giving food to them*. *This tradition goes on to the children of the patients*. *The children of the beggars go begging again*. [BM4, male]

*In the past*, *I had to struggle for food*, *so I had no time to think about education*. *I didn’t go to school or hear about it*, *and I couldn’t send my children to schools either*. [K2, male, leprosy patient with skin patch]

### Context

The lack of accessibility to proper health care and an appropriate social safety net exacerbated the suffering that LAPs had to face.

#### Lack of accessibility to proper health care

Many patients experienced difficulty in getting proper leprosy care. They were not able to be diagnosed with leprosy at other hospitals, or by private doctors. Patients expressed frustration because of the repeated courses of unnecessary investigations, misdiagnoses and ineffective treatment that they had to face.

*When I first had leprosy symptoms and skin lesions*, *I went to a private doctor*. *But he didn’t diagnose me of leprosy*. *I spent a lot of money*, *maybe almost 20000 rupees on treatment that I didn’t need*, *and met 7 to 8 private doctors*. *I had to suffer for more than one year before coming to Little Flower Hospital*. [P3, male, leprosy patient with body disfigurement]

*The first time I got ulcers*, *it spread very quickly because of improper care at another hospital*. *It was a small wound initially*, *but in two to five days*, *it became bigger and bigger*. [P4, male, leprosy patient with body disfigurement]

#### Lack of social safety net for the protection of LAPs

The patients desire to live a life with fulfilled needs, but the support provided by the government does not meet the societal need of the patients.

*To solve the leprosy problem*, *government says that it is just a disease and wants to reduce the number of disease*. *So they only supply clinical support*. *They provide medicine*, *and that’s it*. *This is where the problem is*. *The supply doesn't meet the patient’s demand*, *and this is the reason why leprosy disease still persists for a long time*. *… The government says ‘I give you this amount of free medicine*, *and you adjust with that*.*’ And whatever they have given*, *has not alleviated suffering*. [BM1, female]

### Condition

Even with the aforementioned contingencies and context, willingness of a better life doesn’t lead straight to the success of this hospital. There are specific conditions that made the consequences possible.

#### Bottom-up formation of a consumer-provider cooperative

In LFH, there are no professional doctors to treat patients, only a few nurses and physiotherapists. However, patients mutually provide basic medical services to treat each other every day. They learned the methods of ulcer treatment from nurses and trained themselves. Patients who work as dressers for ulcer care are not professionals, but they provide appropriate care to more than 50 admitted patients every day. In addition, other non-medical services of the hospital are also provided by the efforts of LAPs. They work as cooks to distribute food to admitted patients, and cleaners to maintain good sanitation of the hospital.

*I came to Little Flower Hospital Community 37 years ago*, *at 1981*, *when this hospital was built*. *At that time*, *I had leprosy*, *so I had to amputate my leg*. *Since then*, *I am working in the hospital as a dresser*. *… One time*, *during my job*, *I cleaned another patient’s ulcer*, *and it healed*. *He thanked me a lot*, *and I was satisfied with my job*. [P9, male, leprosy patient with amputation]

*I came to this hospital for leprosy treatment but started to work as a cook in the hospital to provide food to patients*. *I have been working for 20 years*. *… I go to the hospital at 4 am in the morning*, *break the coal*, *make fire*, *and cook rotties and rice to eat*. *I am now deputed to cook*, *but for the Little Flower Hospital Community’s needs*, *I am happy to work anywhere*. [P8, male, leprosy patient with bone contracture]

*The people who are working in Little Flower Hospital are all leprosy patients*. *All who are either affected now*, *or previously affected*. *Only these people and their family can work*. *This is social rehabilitation*. *Except the nurse*, *every worker is a patient*. *The cleaning*, *food making*, *dressing are all done by the patients or their family*. [BM2, male]

Furthermore, in the LFHC surrounding LFH, patients and their family are employed to have economic power which they were not able to have before coming to LFHC. LAPs work in dairy farms making milk, in weaving units making scarves, in wound patch manufacturing factories making bandages, in agricultural sectors harvesting food and in fishery sectors catching fish. The profits gained by selling milk, scarves, patches, food, and fish, are the source of living income for LAPs. Wage is not the only compensation for LAPs’ work. At the same time, the costs needed for providing suitable services in this fulfilling society, are covered by the profit earned by occupational efforts of LAPs. In this way, LAPs are consumers as well as providers of services such as children’s education, clinical treatment including prescribing medication and wound dressing, and electricity in housing simultaneously, and this has been possible through the work that they do. LFHC was able to sustain itself for more than 35 years without massive funds from the government or external resources as an economically self-sufficient cooperative. LFHC does operate, at times, by using their emergency funds which was in place since the foundation of this community. However, most of the financial resources that are used to sustain the community come from selling products and providing services.

*I was born in Little Flower Hospital Community*, *because my mother and father had leprosy*. *After being an adult*, *I started to work in Khadi (weaving unit)*. *… Now I have worked here for 20 years*, *and because of my experience*, *I am in charge of scarf making*. *Every day*, *I distribute the weaving to other workers*, *and my responsibility is to do the dying*, *coloring*, *tailoring*, *and sending it to consumers in the world*. [K1, female, family of leprosy patients]

*I was suffering because of leprosy*, *when I got to know Little Flower Hospital Community*. *After coming here*, *I started to work here in the dairy farm*. *I go to the farm every day 5 am in the morning*, *and provide food and water to all the cows*. *Also*, *I milk the cows and transport the milk to where it is needed*. *… I like my job because through my job*, *I can fulfill my needs*. [D2, male, leprosy patient with amputation]

The theme of *a strong devotion to work for the leprosy community* and *dedication to help other people* continuously emerged from interviews. LAPs were willing to volunteer for helping other patients and thus became the ones who made a difference in each other’s lives.

*When I am doing social work*, *I feel very good*. *I was a leprosy patient myself*, *and when I provide services to other patients*, *I feel internally satisfied*. *I like helping leprosy patients*. *… At the first time I was deputed of another job for farming*, *but I wanted to meet the patients and help them*, *so I requested for a dresser*. [P9, male, leprosy patient with amputation]

*My mother and father are both leprosies*, *so I feel that I want to help*. *… If I work for the hospital*, *it is good for me and my family*. *If I work for leprosy patients*, *it is also good for us*. *Because people have stigma with leprosy*, *I want to help defeat it*. [W2, male, family of leprosy patients]

*To make Little Flower Hospital Community*, *there is a story of struggle*. *All of this that you can see*, *is the sweat of leprosy affected persons*. *Without fingers*, *with wounds and ulcers*, *leprosy affected persons worked to make this community*. *It was not built by money*. *This was built by the effort of leprosy affected persons*. *The history of Little Flower Hospital Community shows the passion of changing life*, *and this is why Little Flower is unique*. [BM1, female]

#### Social rehabilitation through holistic care

Unlike other hospitals or communities, LFHC provides holistic support which takes care of LAPs’ lives in a wholesome aspect. The board members had a vision of working for the patients’ social transformation and mental well-being. Holistic care largely included four components: psychosocial, educational, medical and residential support.

***Psychosocial support to defeat stigma***

The psychosocial care in the form of counseling is given to patients by nurses and physiotherapists, and patients in the same position provide social support to each other. This showed a positive impact on the mind of the patients. Many patients suffered immensely due to the disease’s social stigma and thus experienced feelings of depression. However, many patients expressed that their lives are much better after they came to LFH and that they now have dignity in life.

*Little Flower Hospital motivated me to live a better life*. *If there was no Little Flower Hospital*, *god would have abandoned me*. [P7, male, leprosy patient with body disfigurement]

*If Little Flower did not exist*, *I would have been buried under the ground*. *I would not want to live*, *so I would have stopped eating and avoid food and water to die because of the hardships that I faced*. [D2, male, leprosy patient with body disfigurement]

*After coming to this hospital*, *I feel happiness*, *and I am mentally prepared and boosted up so that I can talk to any other people*. *Many of my problems are taken care of by the hospital*. *… My best memory here is when I talked to another person for the first time at this hospital*. *Everyone is friends here*. [P1, female, leprosy patient with amputation]

***Educational support to cease the intergenerational continuation of poverty***

Education had not been a feasible option for LAPs or their children before coming to LFH because of the stigma from the community. LFHC provides tuition free education and dormitories for children that are not living near Sunderpur. Many of the parents were proud that their children, who were former students of the school of LFH, are living successful lives out of LFH.

*My children got their first education here in Little Flower*. *Then*, *they went elsewhere to get higher education*. *They were very happy with the education they received*. *Now my child is admitted to an MBA program in Bangol* [K1, female, family of leprosy patients]

*I didn’t get good education only until 10*^*th*^ *class*. *But I have two sons and they got very good education here and now they are getting education outside*. *Older one is in Bangol*, *and he is getting his basic nursing education there*. [W1, male, family of leprosy patients]

***Medical support to prevent disabilities***

Leprosy is a disease that requires continuous care even after recovery. Ulcers can be very excruciating, and improper care of ulcers can lead to deformities. LFH prescribes medication and provides rehabilitation free of cost, as a hospital specializing in leprosy care. Physiotherapists examine the whole body of admitted patients looking for new ulcers, so that prompt treatment can be conducted. Physiotherapists educate patients to perform daily self-care which prevents the complete paralysis of arms or legs. Also, LFH pays for the referral costs of the patients. When patients need advanced treatment that LFH cannot provide, they are sent to nearby general hospitals for treatment and LFH pays for this treatment of the condition beyond leprosy as well.

*There was nowhere for me to get treatment*. *In order for me to get treatment and survive*, *I needed to pay money*. *So*, *I came here because treatments were provided free of cost*. [P8, male, leprosy patient with bone contracture]

*Little Flower is very helpful because in the past*, *when I had many troubles regarding health*, *I was not able to go outside to get treatment*. *I didn’t not have money*. [K1, female, family of leprosy patients]

*If Little Flower Hospital was not here*, *there would have been no place for leprosy patients to get proper treatment*, *and people would have died*. *As you might have seen*, *the government hospital in Bihar does not have ulcer units like Little Flower Hospital*. *Also*, *in these hospitals you have to pay*. *But our hospital is free*. [BM3, male, family of leprosy patients]

*If some leprosy patients come from outside and is admitted in our hospital*, *and when they have a problem during that period due to disease other than leprosy*, *they are sent to another hospital for treatment*. *The costs incurring in that hospital is covered by Little Flower*. [W1, male, family of leprosy patients]

***Residential support for the abandoned***

In the past, many LAPs, when they were diagnosed with leprosy or were left with the symptoms of leprosy, were chased out of their community and were abandoned without a place to go. Knowing that such difficulty existed before coming to LFHC, it provided free housing to LAPs.

*Yes*, *I was provided with a house by the founder [of the LFH] and if I didn’t have a house*, *I would not have survived*. *I did not have money to buy another house*, *so I was very very happy*. *… I live a very comfortable life here and I can survive here*. *Before*, *the society planned to kill me*. *Here*, *I am happy*. [P9, male, leprosy patient with amputation]

### Consequence

#### Economical sustainability

Milk made from dairy farms are sold to the general community outside of LFHC, and scarves made by the patients are also sold to many countries in the world. With the profit earned, the hospital supplies raw materials such as cow food and cocoons, and also supplies basic clinical equipment needed for treatment in the hospital. This process is able to be sustained because there is a continuous demand of milk produced by this community, regarding the milk-tea drinking culture of India. Also, LFHC has succeeded in creating a sustainable global market for scarves as well, providing good quality scarves in a reasonable price.

*In India there is a huge demand of milk*. *This is a guarantee*. *People in India love tea*, *especially milk tea*. *The only problem is that they are not provided fresh and good milk*, *but Little Flower Hospital Community can provide that*. *Also*, *European countries buy the scarves to wrap their neck*. *The cotton made scarves are of huge demand*. *A social market is created*. [BM1, female]

*Milk is needed for everybody*, *from the poor to the rich*. *So*, *there is always a market in local*. *Also*, *Mahatma Gandhi was the one who started to make scarves out of cocoons in India*, *as part of a nonviolent production*. *This is one of the successful factors of Khadi (scarf making)*. [BM3, male, family of leprosy patients]

Patients provide services to other patients in the hospital, and LAPs are employed as main laborers of the community. LAPs were pleased to gain economic power and earn money for themselves and their family. Some LAPs were satisfied with their jobs because of the fact that they can spend time with their family while working.

*It is a very good chance and opportunity for me to do something for Little Flower Hospital Community*, *because I am also living here*. *If I earn money and support for Little Flower*, *I will receive the benefit*, *too*. *My wife is also working here*, *and she will get benefit*, *too*. *We are all getting support from Little Flower Hospital Community*, *so our first duty is to think about what is good for this community*. [W1, male, family of leprosy patients]

*When I started working*, *I was feeling good because I was able to earn money*. *I want to continue working for my whole life*, *because I like my job that much*. [K1, female, family of leprosy patients]

*I don’t have a lot of experience for working*, *and I am not a trained person*. *But I try to do any work needed for Little Flower Hospital*. *I feel that it is good for me to work here because I can see my mother and family*. [W2, male, family of leprosy patients]

#### Social sustainability

Life was very difficult before coming to the LFHC due to stigma and poverty, but this changed due to the wholesome support given by LFHC. They were given individual income with employment, education opportunities, appropriate treatment and descent housing. Most of the patients could not imagine the absence of LFH because there would be massive suffering and pain.

*My life is much better after coming to Little Flower Hospital Community*. *My son and daughter is learning here*, *and I have a job of teaching children*. *… I changed a lot*. *I can bring light to people*. *I became strong*, *and leprosy is gone*. *My life has joy now*. [D1, male, leprosy patient with skin patch]

*Little Flower Hospital has been continuing to provide services in a regular basis*. *It always thinks about us*. *If services stop*, *more people will suffer because no other places provide this kind of service*. *… If Little Flower Hospital Community was not here*, *we leprosy people would have been in the roadside*, *developing ulcer*, *not getting proper care*, *and would have died*. [P9, male, leprosy patient with amputation]

Anyone who is a leprosy patient or a family of a patient is given a chance to make a valuable contribution to the community.

*In Little Flower Hospital Community*, *all the people can earn money and feel happy and have a good life*. [D3, male]

*LFH is always helping the leprosy patients from anywhere*. [W2, male, family of leprosy patients]

*In God's eyes*, *everybody is equal*, *so leprosy patients must also be equal in society*. *This organization has educated the society that fact*. *It tries to educate that leprosy is not a genetic disease*. *And now*, *the society is changing*, *and people are being treated okay and not being discriminated*. [BM4, male]

*Little Flower is not like other NGOs*, *or other hospitals*. *Little Flower is just like our house*. *We all come*, *and we all work together and this makes us very happy*. *We all work together as a community*, *like a ‘family’*. [K1, female, family of leprosy patients]

*It’s altogether like a family in Little Flower Hospital Community*. [K3, male, family of leprosy patients]

*I like to work because I can work together with perfect people*. *We work altogether to solve problems helping each other*. [K2, female, leprosy patient with skin patch]

### Covariance

Board members continuously emphasized the importance of education by rhetorically mentioning that they do not wish the next generation of leprosy patients to live in LFHC. Providing basic education allows the children to be employed outside of LFHC, thus inspiring them to live outside of LFHC in a general population.

*We provide services for leprosy patients and their family*, *but there are not much resources to provide everything to the 2*^*nd*^, *3*^*rd*^, *and 4*^*th*^ *generation*. *… Now we are able to manage*, *but slowly*, *slowly I hope the next generation can move out to the society*. *This is why we have an education system*. *We give best education and then children can go outside to earn money*. *Then there is the end of social stigma*. *For example*, *my son*, *a family of leprosy*, *got advanced education and was able to earn money so he did not stay in LFHC*. *If this happens*, *the burden of LFHC will slowly decrease*, *and then*, *‘leprosy’ will be finished*! [BM3, male, family of leprosy patients]

*Children should have high education*, *and they should become part of the mainstream society*. *Hopefully we can grow a hero in the mainstream society*, *not a hero only in our colony*. [BM1, female]

## Discussion

The fundamental cause that induced the sustainability of LFHC was the LAPs’ strong craving to live a better, happier life with dignity, despite the harsh circumstances they were forced to live in. The aspiration to make a difference in life was deeply rooted in their earnest words. The contingency that triggered the LAPs’ desire for a good life was the hostile society that prevented them from living a life with dignity. The themes of *stigma* and *poverty* continuously emerged, showing the harsh situations that LAPs had to face. These important themes were often associated with discrimination and alienation, which restricted LAPs from fulfilling their needs of living. Most of the LAPs coming to LFH have suffered both mentally and physically due to *stigma* from the society or sometimes from their own family. This discrimination led to self-stigma and denial of their own existence, frequently implied within the theme of *suicidal behaviors*. *Poverty* was another important theme that emerged, aggravating the situation by forming a intergenerational cycle continued by malnutrition and weak immunity [21, 22]. Along with the contingencies, contextual factors of the society in Bihar and India made circumstances even more excruciating for the LAPs. The number of experts that used to know how to diagnose and treat leprosy has decreased in a rapid pace, and the accessibility to proper health care for patients is disappearing with the knowledge of leprosy. Furthermore, there is still a lack of social safety net to rescue them from destitution and inequality. Rather than just medical care, LAPs needed a stigma-free society to live in.

With the context of the society not providing the suitable resources for a societal life with dignity, LAPs started to work by themselves for themselves with their own power to build a community that ensures a good life. The community that they established was a form of a ‘consumer-provider cooperative’. The consumers of services and providers of services are not separated. Rather, the action of consuming and providing is held by the same patients. This is how LFHC has been able to sustain itself for more than 35 years without massive funds from the government or external resources, establishing a form of an economically self-sufficient cooperative. For this self-sustainable consumer-provider cooperative to be established through a bottom-up approach, devotion of the LAPs to do social work was essential. Their emotional attachment to LFHC and the passion for change were the essential conditions that induced the bottom-up establishment of the cooperative community. Along with the LAPs’ own efforts of building a cooperative community, the theme of *holistic care provided by the LFHC* was also considered as an important condition. This played a role to socially rehabilitate LAPs to become included as a part of the community. Due to the psychosocial support from LFH, LAPs were freed from a life of suffering where they had to worry about their survival every day. Now that their lives were made easier, they were encouraged to continue their lives. Education was another form of rehabilitation that the hospital provided. Children are socially rehabilitated to develop their own skills and given the possibility to escape the constraints of leprosy. LFH also provides medical and residential support, leading to a fulfilled society that LAPs can live in.

Through the synergistic effect of cause, contingency, context, and most importantly conditions, LFHC has economically and socially sustained itself to provide continuous, regular support to the patients, despite the decrease of funding from the government. Whereas the patients themselves were the ones who made this model possible, the hospital served as an underlying platform of a bottom-up approached sustainable community, in which the supplies have matched the demands of the LAPs. The first condition, *Bottom-up formation of a consumer-provider cooperative* contributed to the economical sustainability of LFHC. The three pillars of production—capital, labor and land—were all shown to be sustainable due to this condition. The component of capital including raw materials necessary for production is sustained by the profits gained through the LAPs’ occupational efforts. The constant demand of the goods produced in LFHC contributed to the economical sustainability of LFHC. The component of labor has been sustained through the mutual cooperation between LAPs. The reasons for the continuous presence of labor appeared to be *devotion to work for the leprosy community* and *satisfaction with jobs*. Even with a very low salary, the labor was able to be sustained due to the LAPs’ dedication and emotional attachment to LFHC. Because LAPs were each given a role in the community, they worked earnestly for the community, and this was the core of economic sustainability. Land, which is the last component of production, was able to be sustained until now because the hospital was built using abandoned land of India’s borders.

Social sustainability could be explained through the second condition, social rehabilitation through holistic care. This condition induced the three themes which contribute to social sustainability: *satisfaction of the basic needs*, *social justice through equal opportunities*, *and social coherence*. The holistic care provided by the LFHC succeeded on fulfilling the basic needs of the LAPs, leading to increase in *satisfaction* among LAPs about their quality of life. *Social justice* was established through the provision of equal opportunities without discrimination, empowering LAPs to participate in the society. Finally, the theme of *social coherence* appeared to sustain LFHC socially. Due to the sense of belonging, different characteristics of leprosy patients and their family from many places throughout India could integrate and form an inclusive society.

The narrow time frame of the sustainability of LFHC only lasts until the burden of leprosy disappears. In broader terms, the wider concept of sustainability embraces the theme of LFHC being *integrated into the society*. Through the gradual integration with the general society, stigma towards leprosy will decrease, finally reducing the suffering of LAPs.

Our study showed the positive impact of forming a consumer-provider cooperative in establishing a sustainable care-giving environment for LAPs. This finding was consistent to another qualitative study conducted in Africa, which aimed to obtain insights into the impact of African economic cooperatives on reduction of poverty [23]. Similarly, another study about health cooperatives in Costa Rica has shown the possibility of cooperatives to provide health services to the entire population even in a low-resource setting [24]. Our research about LFHC has additional meaning in that the research proposes a form of a ‘cooperative community’, including both economic and health services. This result could be considered as a successful, sustainable model of community development using cooperatives, particularly for patients of neglected tropical diseases who are suffering due to isolation from the local community [25].

Another important factor that our study has revealed is the significance of social rehabilitation for building a sustainable community to embrace LAPs. This result was aligned with many guides to leprosy control, as well as strategies from WHO, which emphasized the social aspects of leprosy and community-based rehabilitation [8, 26, 27]. A socio-economic rehabilitation program held in India consistently showed how LAPs were able to achieve well-being through social and economic empowerment with a holistic approach [28]. Beyond social rehabilitation, the three factors of social sustainability that LFHC achieved, satisfaction for basic needs, social justice through equal opportunities and social coherence were consistently suggested by former studies of sustainable development. Access to the basic human needs necessary for a decent standard of living has been stressed in a myriad of development strategies, including the UNDP’s Human Development Report [29, 30]. Through theories of development as freedom, Amartya Sen argued the importance of opportunities for participation in the society [31]. The sense of social cohesion and community ownership was emphasized through diverse efforts of defining social sustainability [32, 33]. Therefore, research on LFHC sheds light on these specific determinants of social sustainability by uncovering a successful example of each factor.

While Bihar accounts for about one fifth of the leprosy patients in India, other endemic areas such as Maharastra and Uttar Predesh still report continuous suffering from thousands of newly detected cases of leprosy [34]. Areas inside and outside of India may have similar contextual factors with the model of LFHC, in that many of the leprosy patients all over the world live in *isolated* colonies due to *social stigma* and *poverty*, with *a lack of society safety net* and *healthcare resources* [21, 35–37]. Furthermore, patients of other neglected tropical diseases (NTDs) throughout the world, such as schistosomiasis, lymphatic filariasis, and leishmaniasis suffer similar situations with LAPs in India [38]. The patients are ostracized from the community, without a life of dignity and opportunities for social participation [39]. These similar contextual situations present the inextricable association within NTDs, stigma, poverty and lack of social inclusion, which connotes the need of an innovative strategy towards sustainability such as the one of LFHC.

There are several limitations that needs to be taken into consideration while understanding the results. First, the fundamental limitation of a Grounded Theory study is that the resulting theory’s transferability is low. This is because the codes, categories, and themes were developed from the words of people with a highly specific background. The limitation of methodology makes it hard to implement the resulting theory in other situations, even if the results can be used for reference. Second, study participants were made to talk about an event that happened in the past (going to the hospital), which could have led to recall bias. Third, social desirability bias may have occurred. Since many of the study participants were not familiar with foreign interviewers, there are possibilities that interviewees answered in ways desired by the society. Fourth, interviewers did not actively attempt to have the interview transcripts reviewed by with the participants, as majority of the interviewees were illiterate. Thus, the content of the interviews was reviewed by three researchers to check the preciseness of the transcript. Finally, interviews were conducted by three researchers from Korea who were not able to communicate with the local people using their language. Even though an interpreter always accompanied the interviews, there might have been misinterpretations of wordings in the translation process.

## Conclusion

This Grounded Theory based study explored the determinants that made LFHC sustainable, through a qualitative research with 28 interviews. The results provided insight into the thoughts of LAPs and the societal context surrounding them which led to a self-sustaining community. This bottom-up approached sustainable leprosy community establishment have been possible through the formation of a cooperative community combined with social rehabilitation.

The findings presented may suggest that this sustainable development model of LFHC is promising and deserves further research. More exploration on transferring this model to other leprosy colonies will have great impact in maintaining sustainable care for leprosy patients. Furthermore, this research may advocate the establishment of a cooperative community and implementation of social rehabilitation in and out of India, by highlighting the importance of sustainable development in policies targeting neglected tropical diseases including and beyond leprosy.
